# Evolution of Phase, Microstructure and ZrC Lattice Parameter in Solid-solution-treated W-ZrC Composite

**DOI:** 10.1038/s41598-017-06301-0

**Published:** 2017-07-26

**Authors:** Peng Jia, Lei Chen, Jiancun Rao, Yujin Wang, Qingchang Meng, Yu Zhou

**Affiliations:** Institute for Advanced Ceramics, School of Materials Science and Engineering, Harbin Institute of Technology, Harbin, 150001 China

## Abstract

Zirconium carbide (ZrC) reinforced tungsten (W) composite was hot-pressed at 2200 °C for 1 h in vacuum, which was subsequently heat treated in the temperature range of 2200 to 2500 °C for 1.5 or 2 h. The relative ratios of ZrC phase were 21.0, 23.3 and 25.9 mol.% for the mixture of starting powders, composites sintered for 1 h and solid-solution treated at 2500 °C for 1.5 h, respectively. The solid solubility of W in ZrC increased with the increment in heat-treating temperature and time to a maximum value of 18.9 mol.% at 2500 °C for 1.5 h. The lattice parameter of cubic ZrC phase diminished from 0.4682 nm in the starting powder to 0.4642 nm in the solid-solution composite treated at 2500 °C for 1.5 h. This work demonstrated that the relationship between the solid solubility of W in ZrC and the lattice parameter of ZrC is linear, with a slope of −1.93 × 10^−4^ nm/at.%. Overall, more W atoms diffused into ZrC lattice after heat treatment, meanwhile, the previous precipitated nano-sized W dissolved in the supersaturated (Zr,W)C solid-solution, as detected by SEM and TEM.

## Introduction

ZrC is an important material for high temperature applications due to its high melting temperature, high Vickers hardness and excellent corrosion resistance^1–4^. However, as with most ceramic materials, poor fracture toughness and thermal shock resistance reduce its potential utility in multiple applications. A great deal of work has been conducted to improve its mechanical properties and oxidation resistance in past decades, with addition of refractory metals or ceramics, such as W, Mo, Nb, ZrO_2_ or SiC, which was reported to be an effective way^5–12^.

In our previous study, dense W doped ZrC (W-ZrC) composites were fabricated by hot-pressing as well as displacive compensation of porosity (DCP) method^13–16^. The research were mainly focused on the effect of sintering temperature, ZrC content and particle size on microstructure and mechanical properties of W-ZrC composites both at room and elevated temperatures. The mechanical properties of the W-ZrC composites was improved, especially toughness, in comparison with pure W or ZrC ceramics. Additionally, as processing temperature increased, the flexural strength of the W-ZrC composites increased to 1100 MPa, while the strength of pure W was decreased 60% from room temperature to about 300 MPa at 1000 °C^17^.

In addition, XRD and TEM analyses revealed an interesting phenomenon. W diffused into ZrC lattice and there formed (Zr_*1*−*y*_W_*y*_)C_*x*_ (x ≤ 1, y < 1) solid-solutions in W-ZrC composites. The similar results in Mo/Nb-ZrC composites were reported by other researchers^18–25^. Some nano-sized W particles were found inside the grain of (Zr_1−y_, W_y_)C_x_ solid-solution, which were beneficial to mechanical behavior, acting as the intragranular nanocomposites^26, 27^.

However, up to now, intragranular nanocomposites only have been fabricated primarily by mixing the reinforcing nano-additive with the substrate powder^28^. Work has hardly been reported on accessing similar structures via solid-solution processing followed by subsequent ageing to obtain the nano-particle reinforced carbide systems, although it is common in Al or Mg alloys^29–31^.

In the present study, W-ZrC composites with 21.0 mol% ZrC were fabricated by hot-pressing and subsequent solid-solution treatment at different temperatures for different time. Both qualitative and quantitative XRD analyses were performed to identify the final phase constitution of the heat-treated composites. Afterwards the relationship between solid solubility of W in ZrC and temperature as well as time was determined. The exact lattice parameter of ZrC solid-solution was also calculated. The target of this study was to characterize the effect of both the temperature and time on phase evolution and ZrC lattice-parameter variation in the studied W-ZrC composites.

## Results and Discussion

### Phase identification and lattice-parameter determination of ZrC solid solution before and after solid-solution treatment

**Figure 1:**
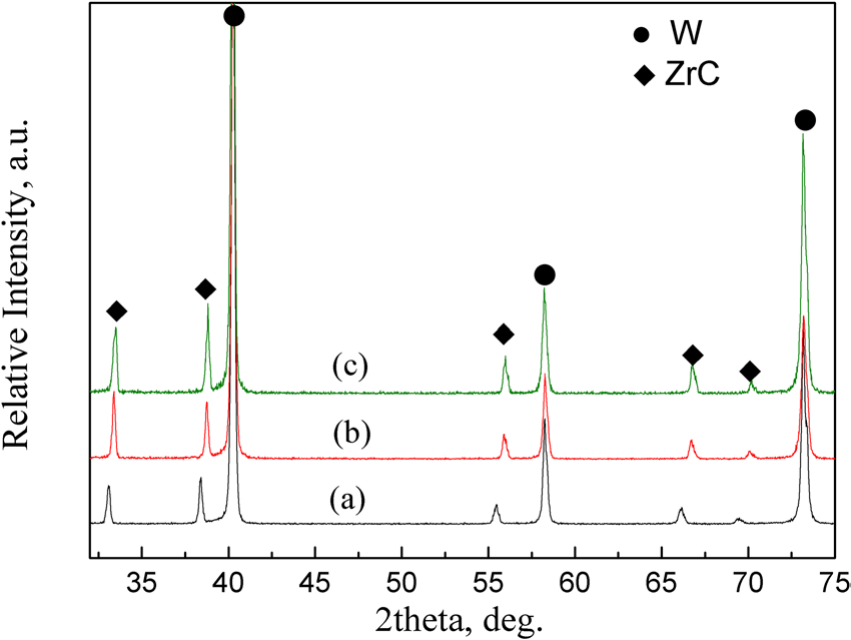
XRD patterns of (**a**) starting powder mixture and composites of (**b**) sintered and (**c**) solid-solution treated at 2500 °C for 1.5 h.

The XRD patterns of the starting mixture of W and ZrC powders, the W-ZrC composites as sintered and after solid-solution treatment at 2500 °C for 1.5 h are shown in Fig. 1(a–c), respectively. Obviously, in Fig. 1a, the diffraction peaks kept the same as the raw materials, showing that there was no new phase formed during ball milling. There are only two phases are detected as W and ZrC, either in the sintered (Fig. 1(b)) or in the heat-treated specimens (Fig. 1(c)), giving XRD patterns similar to those of the starting powder-mixture.

It should be noted that ZrC peaks were shifted slightly to the high angle region in Fig. 1(b and c), while W peaks remain almost identical in the three XRD patterns, compared with that in Fig. 1(a). As demonstrated in Wang’s study^10, 11^, the shift of ZrC diffraction peak comes from formation of (Zr_1−y_W_y_)C_x_ solid-solution, x for ZrC appearing as a nonstoichiometric compound. There is no detectable solubility of ZrC or Zr in W, so the diffraction peaks of W does not shift. After sintering and heat treatment, the ZrC peaks shift toward higher angles indicating a decrease in its d-spacing, equated to the lattice parameter.

**Figure 2:**
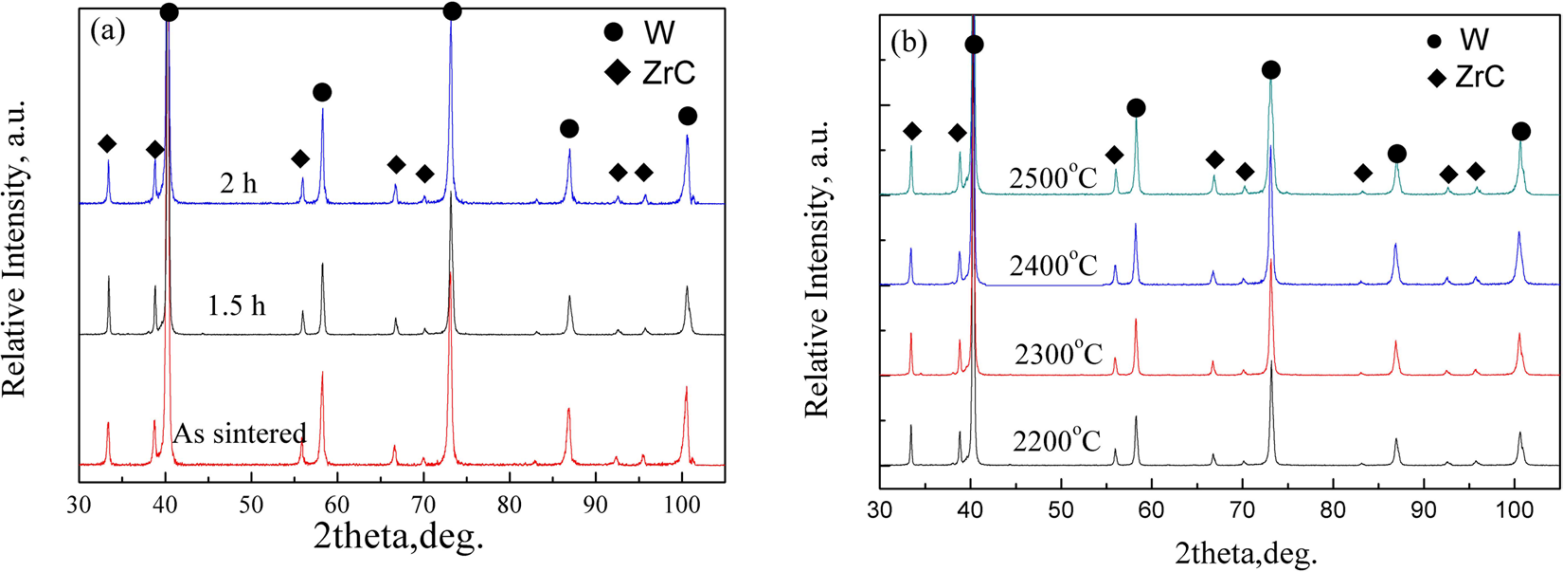
XRD patterns of W-ZrC composites (**a**) solid solution treatment at 2200 °C for different time and (**b**) at different temperatures for 1.5 h.

Effects of solution-treatment temperature and time on the phase constitutions and ZrC lattice parameters in the W-ZrC composites were analyzed by XRD. The results are shown in Fig. 2(a and b). It is obvious that only peaks of the W and (Zr_1−y_W_y_)C_x_ phases are observed, and that the peaks of (Zr_1−y_W_y_)C_x_ solid-solution phase shift slightly to higher angles. The results suggest that the lattice parameter for (Zr_1−y_W_y_)C_x_ solid-solution decreases and the solid solubility of W in ZrC phase increases with increment of the treating temperature and time.

The solid solubility of W in ZrC changes with temperature as shown in the C-W-Zr ternary phase diagram^32, 33^, *i.e*., increasing from ~10 mol % at 2200 °C to ~18 mol % at 2600 °C. More W atoms diffuse into the ZrC lattice with increasing temperature, with a substitutive solid-solution forming. Meanwhile, the covalent radius of Zr (0.145 nm) is larger than that of W (0.138 nm)^34^, so the lattice parameter of the (Zr_1−y_W_y_)C_x_ solid solution should decrease with the increment of solid solubility of W in ZrC. The diffusion of W into ZrC may be also beneficial to composite sintering and densification^19^.

The ZrC lattice parameter was determined according to the Bragg’s law and with a method of linear extrapolation to reduce the error. The lattice parameters calculated were accurate to ± 0.0002 nm. With the increment both of temperature and time of solid-solution treatment, the lattice parameter of (Zr_1−y_W_y_)C_x_ solid-solution decreased slightly, from 0.4682 nm of the starting powder to 0.4668 nm of the as-sintered sample. Table 1 gives the detailed results of the lattice parameter of (Zr_1−y_W_y_)C_x_ solid-solution, clearly showing it decreased from 0.4662 to 0.4642 nm when the treating temperatures from 2200 to 2500 °C. After solid-solution treatment for 2 h, the ZrC lattice parameter decreases 0.0009 nm.

**Table 1:** Lattice parameter of ZrC at different temperatures and for different time.

Sample	W(110):ZrC(111) (wt. ratio)	W(110):ZrC(111) (mol. ratio)	Cal. Value/nm
The Starting Powder Mixture	6.32	3.48	0.46822 ± 0.00013
As Sintered	5.82	3.27	0.46682 ± 0.00008
S22–1.5 (2200 °C, 1.5 h)	5.65	3.18	0.46627 ± 0.00010
S22–2.0 (2200 °C, 2.0 h)	5.55	3.12	0.46592 ± 0.00007
S23–1.5 (2300 °C, 1.5 h)	5.48	3.08	0.46560 ± 0.00009
S24–1.5 (2400 °C, 1.5 h)	5.22	2.93	0.46514 ± 0.00011
S25–1.5 (2500 °C, 1.5 h)	4.93	2.77	0.46428 ± 0.00006

Values are ± 1.6 mol%.

### Quantitative analyses of phase constitution

Up to now, the mathematical relationship between the lattice parameter of (Zr_1−y_W_y_)C_x_ and solid solubility of W in ZrC phase is still unknown. It is instructive to estimate the solid solubility of W in ZrC based on the values of (Zr_1−y_W_y_)C_x_ lattice parameter, and to calculate the exact compositions of the C-W-Zr ternary system.

As shown in Fig. 2a and b, with increment both of the temperature and time of solid-solution treatment, the diffraction peaks of (Zr_1−y_W_y_)C_x_ increase in its intensity while the W peaks decrease. It means that the relative content of ZrC increases incrementally and the relative content of W decreases accordingly with increasing temperature and time. The content of W and ZrC varies due to the formation of (Zr_1−y_W_y_)C_x_ solid-solution. More W diffuses into ZrC when the system is heated. Like the variation of ZrC lattice parameters, the solid solubility of W in ZrC phase increases with increment of treatment temperature and time. The results confirm that (Zr_1−y_W_y_)C_x_ solid-solutions result from diffusion of W into ZrC via the following reaction (written on the basis of 1 mole of ZrC reactant):

1 $$y/(1-y){\rm{W}}+{{\rm{ZrC}}}_{{\rm{x}}}\to (1/1-{y)(\mathrm{Zr}}_{{\rm{1}}-{\rm{y}}}{{\rm{W}}}_{{\rm{y}}}{)C}_{{\rm{x}}({\rm{1}}-{\rm{y}})}$$

Here *y* refers to the solid solubility of W in ZrC. Quantitative XRD analysis was conducted to calculate the values of *y* after sintered and solid-solution treatment. The values of *y* could be derived according to the ratio of W to ZrC at starting mixture, as-sintered or solid-solution-treated state at different temperatures and time.

**Figure 3:**
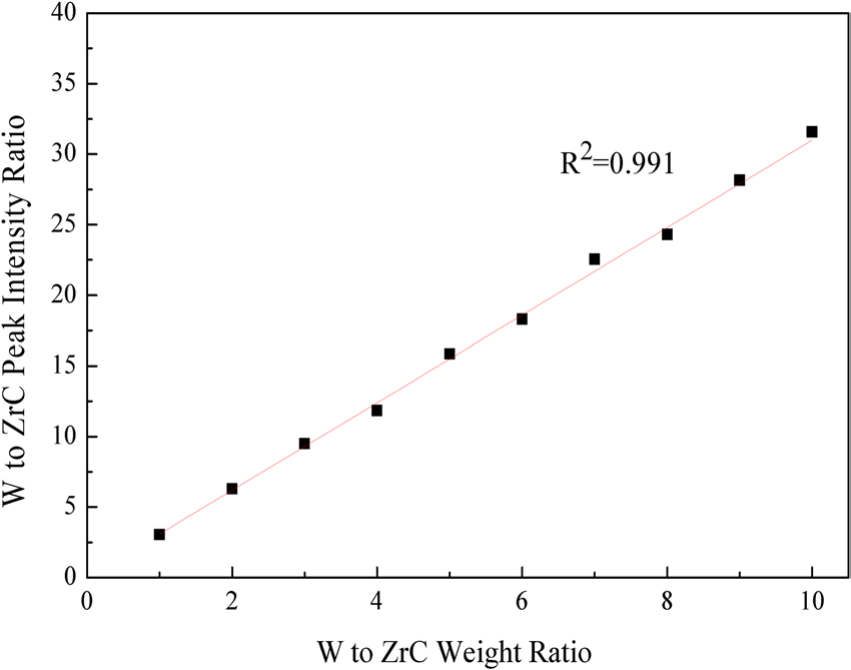
XRD calibration curve derived with the W (110) and ZrC (111) peaks in the mixed powders to determine the content change of W and ZrC phases after solid-solution treatment.

**Figure 4:**
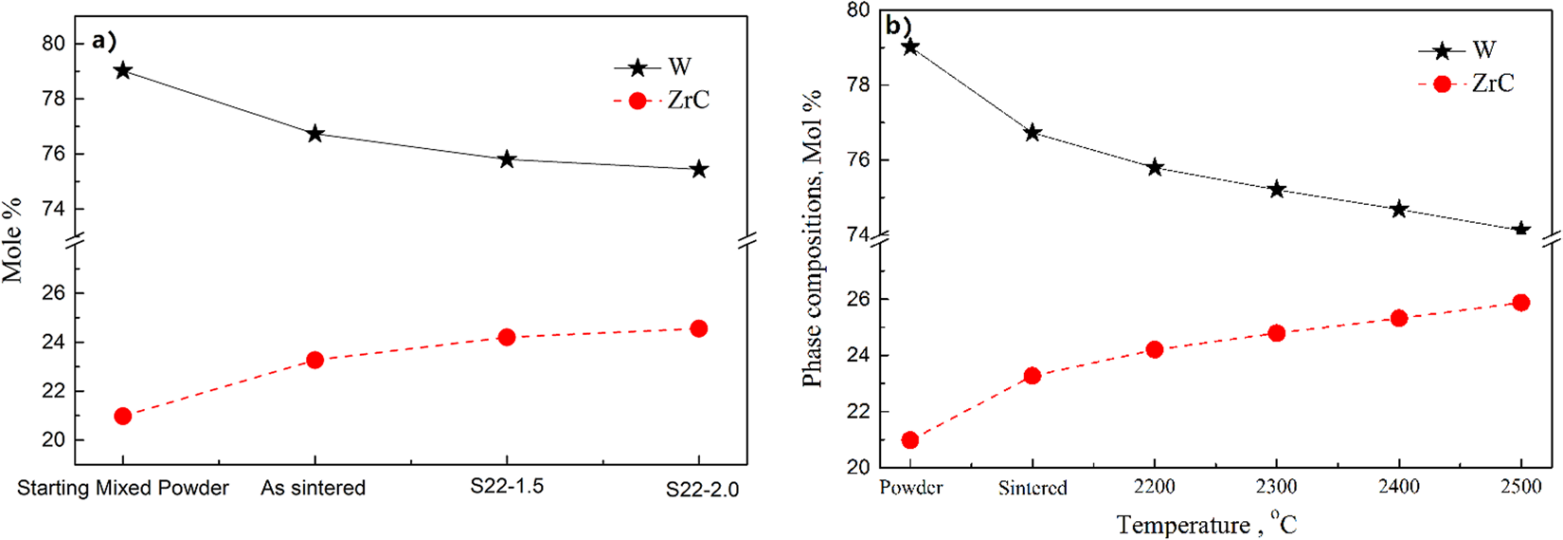
Measured phase compositions of the starting powder mixture and the composites solid-solution (**a**) treated at 2200 °C for different time and (**b**) treated at different temperatures for 1.5 h.

To determine the W and ZrC contents in the solid-solution treated W-ZrC composites, a mathematical relationship was established between intensity ratio of the diffraction peaks and weight ratio. Figure 3 shows the XRD calibration curve used for calculating the contents of W and ZrC. The values of W to ZrC weight ratio were chosen as the horizontal coordinate and values of intensity ratio of (110)_W_ and (111)_ZrC_ peaks as the longitudinal coordinate. The intensity ratio is plotted against the W:ZrC weight ratio for each mixed powder to generate a calibration curve. Then the data were fitted by least-square method. The mathematical relationship between the intensity ratio and weight ratio is linear, with the regression coefficient (R^2^) of equal to 0.991. This calibration curve was used to calculate the constitution of W and ZrC phases in the solid-solution treated W-ZrC composites, with the calculated 1.6% values error. The results are shown in Fig. 4a and b.

Figure 4a is the measured phase constitution in W-ZrC composite treated at 2200 °C for different time (note: time for the as-sintered specimen is denoted as zero). For comparison, the composition of the starting powder mixture is also given in Fig. 4a. The starting powder consists of 21.0 mol% ZrC and 79.0 mol% W. The relative ZrC content increases incrementally with solid-solution-treatment time. The contents are 23.3 mol%, 24.2 mol% and 24.6 mol% for samples treated at 2200 °C for 0, 1.5 and 2 h, respectively. On the contrary, the relative content of W decreases progressively. Solid-solution-treatment temperatures have the similar effect on phase constitution in W-ZrC composite (Fig. 4b). The relative content of (Zr_1−y_W_y_)C_x_ solid solutions increases with increment in solid-solution-treatment temperature, while the relative content of W decreases, and the relative content of (Zr_1−y_W_y_)C_x_ solid solutions with a maximum value of 25.9 mol% when treated at 2500°C for 1.5 h.

**Figure 5:**
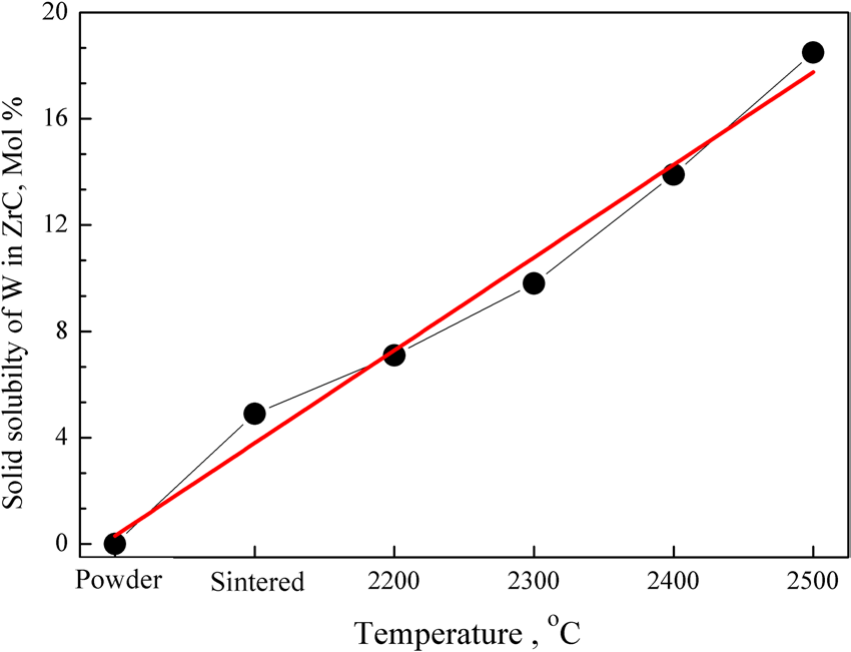
Effect of solid-solution-treatment temperature on solid solubility of W in (Zr_1−y_W_y_)C_x_ solid-solution from 2200 °C to 2500 °C for 1.5 h.

By measuring the ratios of W to ZrC in the solid solution treated at different temperatures and for different time, the solid solubility of W in ZrC (*y* values) could be calculated. As shown in Fig. 5, the solid solubility of W in (Zr_1−y_W_y_)C_x_ solid solutions increased with temperatures, with a greatest values of 18.9 mol%, which was consistent with the results as expected from the phase diagram^32, 33^.

**Figure 6:**
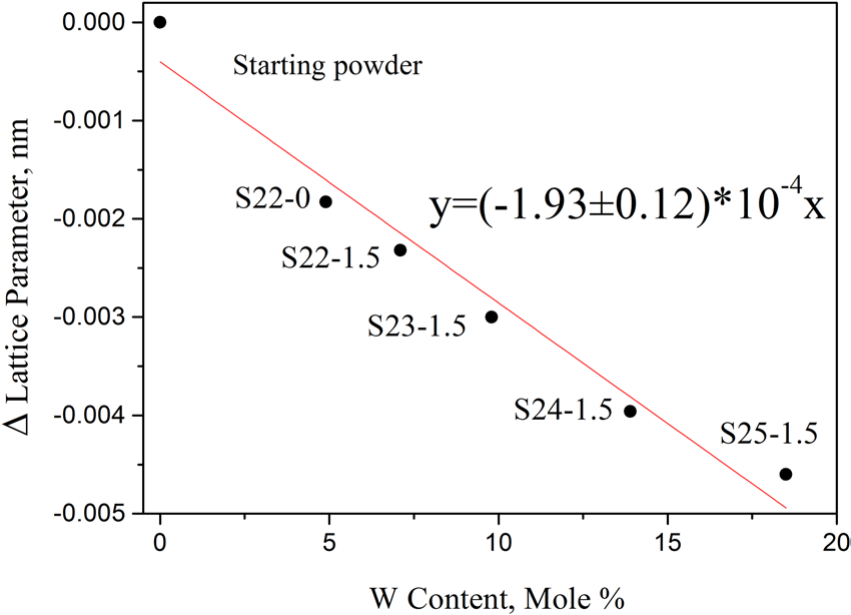
Relationship between solid solubility of W in ZrC and the change of ZrC lattice parameter.

As shown in Fig. 6, the mathematical relationship between the solid solubility of W in (Zr_1−y_W_y_)C_x_ and the change of lattice parameter of (Zr_1−y_W_y_)C_x_ solid-solution was almost linear, *i.e*. the change equals to −1.93 ( ± 0.12) × 10^−4^ nm per at.% W. This result is important in the C-Zr-W ternary system, and rarely reported in other research work.

Nevertheless, the effect of carbon content on lattice parameter of (Zr_1−y_W_y_)C_x_ was ignored in the present study because it was more than 3 times smaller (3 × 10^−5^ nm/at.% C) than that of W^17^. While the effect of W on lattice parameter of ZrC is less than that of Mo (−4 × 10^−4^ nm/at.% Mo)^17, 22^. This is mainly due to different covalent radii (r_W_ = 0.138 nm, r_Mo_ = 0.136 nm) and distortion energy^22, 23^.

### Microstructure of sintered and solution-treated W-ZrC composites

**Figure 7:**
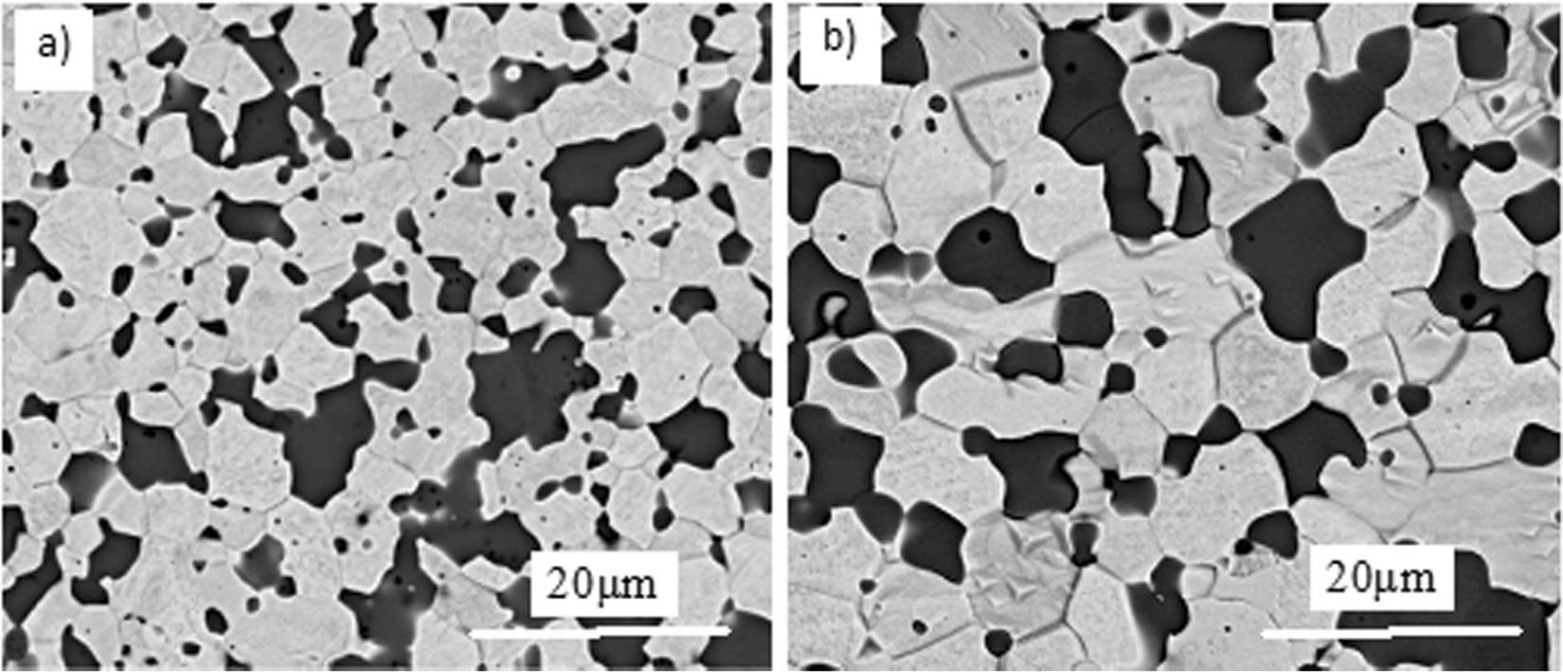
Polished and etched surface of as-sintered (**a**) and 2500 (**b**) solution-treated samples.

SEM micrographs with back scattered electron (BSE) detector of polished and etched surface of as-sintered and 2500 °C solution-treated samples are shown in Fig. 7. W shows brighter contrast, while (Zr_1−y_W_y_)C_x_ solid solution shows darker. W is the matrix phase, and fine dispersed second phase of ZrC particles are well distributed in all over of the matrix, also, some bright rich W phases are detected inside the ZrC grains. Both W and ZrC grains are coarsening with the temperature rising during solid-solution treatment. W grains grow from 5.54 ± 0.28 μm to 10.63 ± 0.49 μm, and ZrC from 3.45 ± 0.29 μm to 5.26 ± 0.25 μm. The different coarsening rates of W and (Zr_1−y_W_y_)C_x_ solid solutions are mainly due to the W diffuses more easily than (Zr_1−y_W_y_)C_x_ phase.

**Figure 8:**
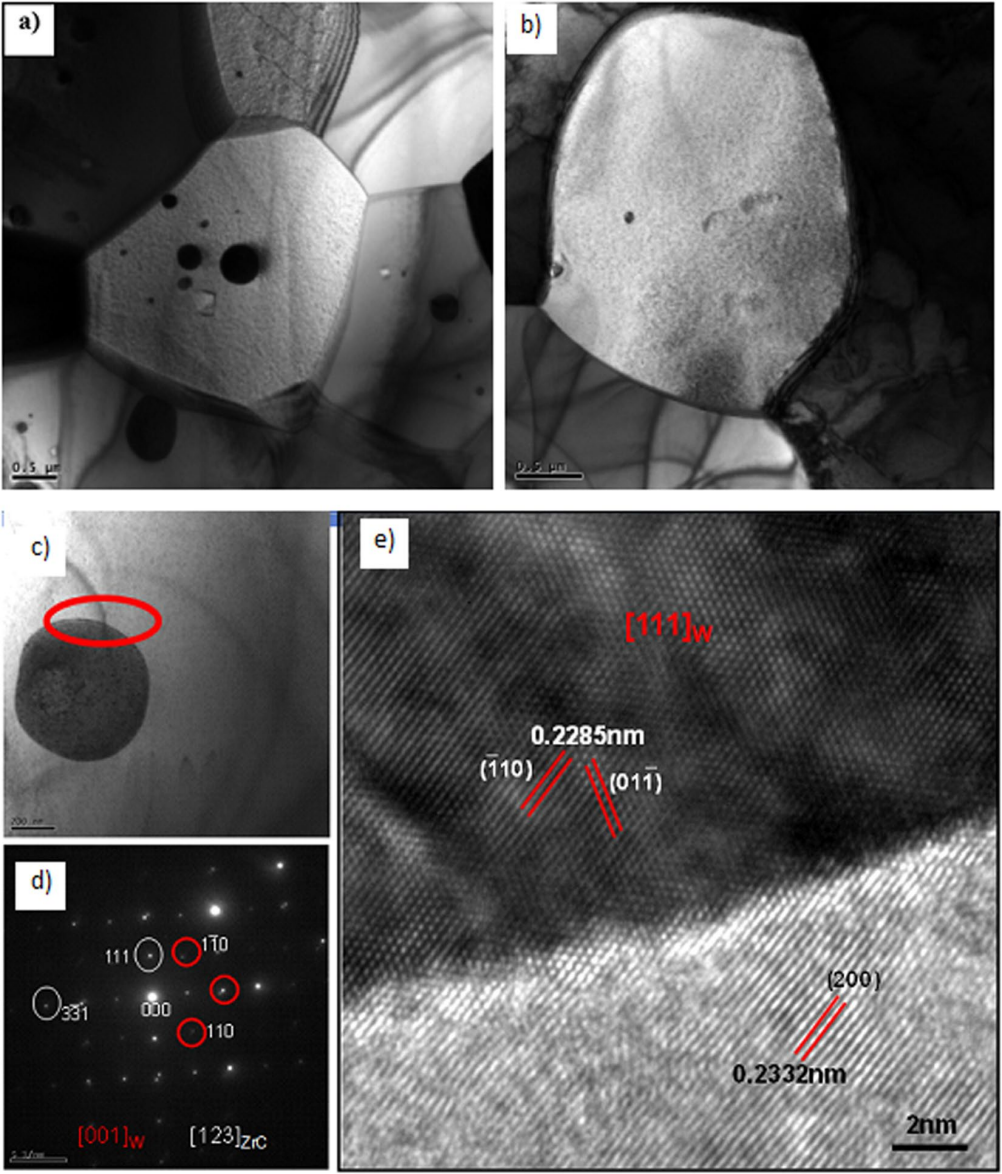
As-sintered: (**a**) TEM images of the composite, (**c**) precipitation phase, (**d**) its corresponding SAED patterns, (**e**) HRTEM of precipitation phase/ZrC interface; 2500°C for 1.5 h: solution-treated: (**b**) TEM images of the composite

Samples of the as-sintered and heat-treated at 2500 °C are characterized by transmission electron microscopy (TEM) technique in Fig. 8. As shown in Fig. 8(a and b), the darker grain is W phase, and the brighter grain is (Zr_1−y_Wy)Cx solid solution. Nano precipitates were detected inside the ZrC grains in the as-sintered sample, as shown clearly in Fig. 8(a). On the contrary, there is no precipitate observed in the 2500 °C heat-treated sample as shown in Fig. 8(b). It indicates that more W diffuses into ZrC lattice during heating and the solid solubility of W in ZrC increases with temperature, which matches the XRD results very well.

Spherical precipitates about 500 nm in diameter are shown in Fig. 8(c). The combined selected area electron diffraction (SAED) pattern both from this precipitate and adjacent matrix was given in Fig. 8(d), which confirms the precipitate is W phase. Figure 8(e) gives the high-resolution transmission electron microscopy (HRTEM) micrograph of interface between the precipitate and the matrix phase^35, 36^. One crystallographic orientation relationship can be deduced from this micrograph, Semi-coherent interface is detected between the W and ZrC phase, as the d-spacing of (110)_W_ is 0.2285 nm, (200)_ZrC_ equals to 0.2332 nm, the results are very closed, with a difference of 2%.

## Conclusions

W-ZrC composites with 21.0 mol% ZrC were fabricated by hot-pressing and subsequent solid-solution treatment at different temperatures for different time. Both qualitative and quantitative XRD analyses were performed to identify the final phase constitution and the lattice of the (Zr_1−y_W_y_)C_x_ solid solutions in the heat-treated composites. Scanning electron microscopy (SEM) and transmission electron microscopy (TEM) are utilized to investigate the microstructure, grain size, the precipitation and relationship between the precipitation and the matrix of the W-ZrC composites. We show that there are two phases of W and (Zr_1−y_W_y_)C_x_ without any new phase formed in the studied composites before and after solid-solution treatment, however, the relative contents of W and (Zr_1−y_W_y_)C_x_ have been differed with temperatures and time. The relative content of ZrC changed from 21.0 mol% in the starting powder mixture to 25.9 mol% in the solid-solution treated W-ZrC composite at 2500 °C for 1.5 h. The lattice parameter of (Zr_1−y_W_y_)C_x_ decreased with solid-solution-treatment temperature and time increasing, from 0.4682 nm in the staring powder mixture to 0.4642 nm in the composite treated at 2500 °C for 1.5 h. The solid solubility of W in ZrC was obtained according to the variation of W and ZrC, with the largest value of ~18.9 mol% at 2500 °C for 1.5 h. The mathematical relationship between the lattice parameter of (Zr_1−y_W_y_)C_x_ solid solution and solid solubility of W in ZrC phase was linear, with a slope of about −1.93 ± 0.12 × 10^−4^ nm/at.% W. The result was reported for the first time, and useful for calculating the compositions in the C-W-Zr ternary system. The nano precipitated phase is W, spherical, and with a size of ~ 500 nm. The crystal plane of (110)_W_ and (200)_ZrC_ matched well. The precipitated phase was demised (or disappeared) after solid-solution treated, it means that the precipitated W diffuses into the matrix phase of ZrC, as the solid solubility of W in ZrC increasing.

## Methods

### Fabrication and solid-solution treatment

Commercial W (99% purity, 3.5 μm average size, Xiamen Golden Egtet Special Alloy Co., LTD, Xiamen, China) and ZrC (98% purity, 1.25 μm average size, Changsha Weihui Special Alloy Co., LTD, Changsha, China) powders were used as the starting materials with a stoichiometry of ZrC_0.965_, according to the analyzed composition results.

A mixture of W and ZrC with the mole ratio of 79.0 to 21.0 was dispersed in ethanol (200 ml) by ball milling (Fe balls, with a weight ratio of 2:1 of ball to powder) in a plastic container (1000 ml) for 24 h. After drying and sieving through a 200-mesh screen, the well mixed powder was placed in a graphite die, and then hot pressed into pellets in Advanced Vacuum System (AVS, model 1540, USA) at 2200 °C for 1 h under a uniaxial pressure of 30 MPa in vacuum. The heating and cooling rates were both set as 20 °C/min.

Pellets were cut into 10 × 10 × 10 mm^3^ for solid-solution treatment. The subsequent solid-solution treatment was performed in a graphite-element furnace in vacuum. The specimens of W-ZrC were placed in a graphite container and heated at 50 °C/min to the target temperature and then held for a specific time. Table 1 summarized the detailed treatment temperatures and time used in this study. The temperatures were 2200, 2300, 2400 and 2500 °C and the holding time varied from 1.5 to 2 h which is the limit of the equipment. After solid-solution treatment, specimens were cooled to 1000 °C within 15 min in furnace in vacuum.

### Phase analysis

Both phase identification (qualitative analysis) and constitution (quantitative analysis) as well as the accurate lattice parameter of (Zr_1−y_W_y_)C_x_ solid-solution in the W-ZrC composites before and after solid-solution treatment were examined by X-ray diffraction (Rigaku, D/Max 2200VPC, Tokyo, Japan) with Cu K_α_ radiation (Ni filter eliminated K_β_) at 45 kV and 100 mA.

For quantitative analysis, a set of W-ZrC standard-powder mixtures, with W to ZrC weight ratios varied from 1 to 10, were used for setting up the XRD calibration curves. XRD data were collected at a scanning rate of 1 °/min from 32 to 42 ° (2θ) to examine the diffraction intensity at the (110)_W_ and (111)_ZrC_ reflections. The amount of metallic W and ZrC in W-ZrC composites after solid-solution treatment was determined by calculating the intensity ratio of these two peaks. Note that specimens for quantitative analysis were ground into powders and sieved, just like the standard-powder mixtures. The change in lattice parameter of (Zr_1−y_W_y_)C_x_ solid-solutions with solid solubility of W in ZrC were determined from the calculated values.

In order to obtain more accurate experimental data, pure silicon powder was added into the samples as an internal standard for system calibration, which precision can be achieved up to ± 0.0002 nm.

All the XRD results obtained were analyzed and refined with Jade 5.0 ^®^ software (MDI, California, USA). An average value was obtained with several measurements.

Microstructure was characterized using scanning electron microscopy (SEM, FEI Quanta 200 FEG) and transmission electron microscopy (TEM, FEI Tecnai F30) equipped with X-ray energy dispersive spectroscopy (EDS). The surface of SEM samples was polished with a series of diamond pastes and finished at 1 μm before etching. To perform an analysis of the microstructure and grain size of the W-ZrC ceramics, some of the samples were etched using a HF: HNO_3_: H_2_O solution, with the volume proportions of 10:10:30 (after being polished using diamond millstones with different particle sizes, successively). Average grain size, its distribution and standard deviation were presented by the software of Image-Pro Plus (ipp 6.1). TEM specimens were prepared through conventional cutting (500 μm thick) and polishing (50 μm), and at last ion-milling at 5 kV (PIPS 691, Gatan Inc., USA).
